# In Vitro Phenotypic Activity and In Silico Analysis of Natural Products from Brazilian Biodiversity on *Trypanosoma cruzi*

**DOI:** 10.3390/molecules26185676

**Published:** 2021-09-18

**Authors:** Raiza B. Peres, Ludmila F. de A. Fiuza, Patrícia B. da Silva, Marcos M. Batista, Flávia da C. Camillo, André M. Marques, Lavínia de C. Brito, Maria R. Figueiredo, Maria de N. C. Soeiro

**Affiliations:** 1 Laboratório de Biologia Celular, Instituto Oswaldo Cruz, Fundação Oswaldo Cruz (FIOCRUZ), Avenida Brasil 4365, Manguinhos, Rio de Janeiro 210360-040, Brazil; raiza.peres@ioc.fiocruz.br (R.B.P.); ludmila.fiuza@ioc.fiocruz.br (L.F.d.A.F.); pbs@ioc.fiocruz.br (P.B.d.S.); meusermb@ioc.fiocruz.br (M.M.B.); 2 Laboratório de Tecnologia para Biodiversidade em Saúde/LDFito, Instituto de Tecnologia em Fármacos (Farmanguinhos), Fundação Oswaldo Cruz (FIOCRUZ), Avenida Brasil 4365, Manguinhos, Rio de Janeiro 210360-040, Brazil; flavia.camillo@far.fiocruz.br (F.d.C.C.); andre.marques@far.fiocruz.br (A.M.M.); laviniabrito@yahoo.com.br (L.d.C.B.); maria.figueiredo@far.fiocruz.br (M.R.F.)

**Keywords:** *T. cruzi*, Chagas diseases, natural products, withanolides, aurelianolides, in silico ADMET

## Abstract

Chagas disease (CD) affects more than 6 million people worldwide. The available treatment is far from ideal, creating a demand for new alternative therapies. Botanical diversity provides a wide range of novel potential therapeutic scaffolds. Presently, our aim was to evaluate the mammalian host toxicity and anti-*Trypanosoma cruzi* activity of botanic natural products including extracts, fractions and purified compounds obtained from Brazilian flora. In this study, 36 samples of extracts and fractions and eight pure compounds obtained from seven plant species were evaluated. The fraction dichloromethane from *Aureliana fasciculata* var. *fasciculata* (AFfPD) and the crude extract of *Piper tectoniifolium* (PTFrE) showed promising trypanosomicidal activity. AFfPD and PTFrE presented EC_50_ values 10.7 ± 2.8 μg/mL and 12.85 ± 1.52 μg/mL against intracellular forms (Tulahuen strain), respectively. Additionally, both were active upon bloodstream trypomastigotes (Y strain), exhibiting EC_50_ 2.2 ± 1.0 μg/mL and 38.8 ± 2.1 μg/mL for AFfPD and PTFrE, respectively. Importantly, AFfPD is about five-fold more potent than Benznidazole (Bz), the reference drug for CD, also reaching lower EC_90_ value (7.92 ± 2.2 μg/mL) as compared to Bz (23.3 ± 0.6 μg/mL). Besides, anti-parasitic effect of eight purified botanic substances was also investigated. Aurelianolide A and B (compounds **1** and **2**) from *A. fasciculata* and compound **8** from *P. tuberculatum* displayed the best trypanosomicidal effect. Compounds **1**, **2** and **8** showed EC_50_ of 4.6 ± 1.3 μM, 1.6 ± 0.4 μM and 8.1 ± 0.9 μM, respectively against intracellular forms. In addition, in silico analysis of these three biomolecules was performed to predict parameters of absorption, distribution, metabolism and excretion. The studied compounds presented similar ADMET profile as Bz, without presenting mutagenicity and hepatotoxicity aspects as predicted for Bz. Our findings indicate that these natural products have promising anti-*T. cruzi* effect and may represent new scaffolds for future lead optimization.

## 1. Introduction

Chagas disease (CD) or American trypanosomiasis is a neglected disease that affects more than 6 million people worldwide with about 75 million people under risk of infection [1]. Currently, benznidazole and nifurtimox are the only available medicines for CD. Both are old drugs only effective in the early stages of the infection, as treatment of chronic patients has low and variable efficacy [1,2]. Another important concern is the high incidence of adverse effects that compromise the continuity of treatment, leading to about 20–30% therapeutic abandonment [3]. In the last years, although advances have been achieved in pre-clinical tests, very few compounds moved to clinical trials, and up to now, no novel drug is available [4,5,6,7]. Thus, new therapeutic options are needed and research on natural products from different sources (e.g., land, sea, plants, microbes and animals) can provide novel chemotype motifs with antiparasitic properties. In this regard, botanic diversity including a wide range of extracts, fractions, and purified substances has been investigated in phenotypic studies searching for new biomolecules presenting anti-*T. cruzi* activity [8,9,10]. 

In this scenario, our present study screened the toxicity and anti-*T. cruzi* activity of 36 plant extracts and fractions (Tables 1 and 2) besides 8 purified substances (compounds **1**–**8**, Table 3) obtained from Brazilian flora composed by *Aureliana fasciculata* var. *fasciculata*, *Clusia studartiana*, *Crescentia cujete*, *Malpighia glabra*, *Piper tectoniifolium* and *Rheedia longifolia* samples. These natural products were selected based on previous literature that reported several pharmacological activities related to these plant samples: (i) leaves extracts from *Aureliana fasciculata* var. *fasciculata* (Solanaceae) have promising activity against *Leishmania amazonensis* [11]; (ii) extracts from leaves and stem bark of *Crescentia cujete* (Binoniacea) have anti-inflammatory and antibacterial in vitro activities; possibly related to tannins, flavonoids, flavones and flavonols [12]; (iii) extracts from *Clusia* (Clusiaceae) species and *Malpighia glabra* (Malpighiaceae) rich in carotenoids and flavonoids present potent antioxidant activity [13,14,15]; and (iv) aqueous extract pf *Rheedia longifolia* (Clusiaceae) leaves has analgesic and anti-inflammatory in vivo activities and low toxicity [16]. Additionally, natural products from Piper species (Piperaceae) display broad biological roles [17,18,19,20,21], including antiprotozoal effects [22]. Additionally, a chalcone isolated from inflorescences of *P. aduncum* [23] and an essential oil and the ethanolic extract from leaves of *P. marginatum* [24] also present promising in vitro effect against *L. amazonensis*. 

## 2. Results and Discussion

### 2.1. Crude Extracts and Fractions

**Figure 1:**
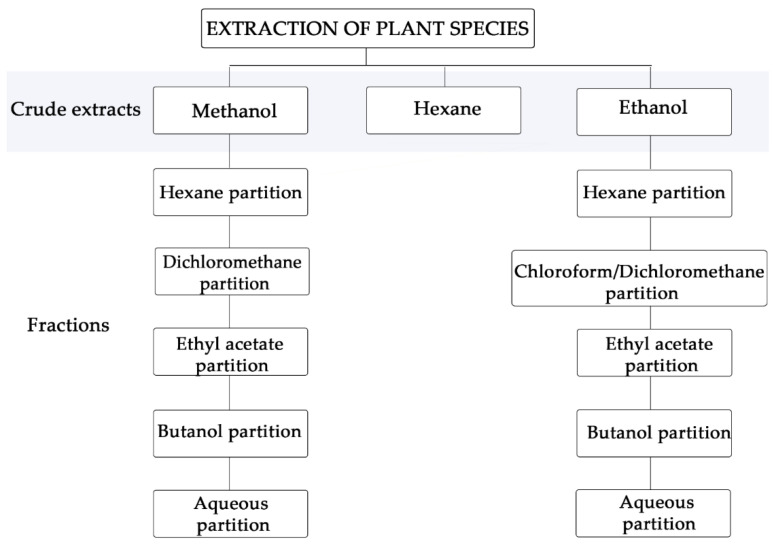
Extraction scheme of extracts and fractions from Brazilian botanic sources.

Initially, the natural products from *Aureliana fasciculata* var. *fasciculata*, *Clusia studartiana*, *Crescentia cujete*, *Malpighia glabra*, *Piper tectoniifolium* and *Rheedia longifolia* were obtained. A guided extraction approach was performed from leaves, fruit pulp or inflorescence, and submitted to partition using solvents with crescent polarities (Figure 1). 

The crude extracts and the resulting fractions were evaluated following a well standardized protocol of phenotypic analysis for novel drug candidates for CD [25]. The first approach was the screening against intracellular amastigotes (Tulahuen-β gal strain, DTU VI) using a fixed concentration of 10 µg/mL, which corresponds to the 90% effective concentration (EC_90_)value of the reference drug (Bz) for CD (Table 1). The leaves fraction using dichloromethane from *Aureliana fasciculata* var. *fasciculata* (AFfPD) sterilized *T. cruzi* infection in L929 cell lines, leading to 100% of infection reduction. Crude extract of *C. studartiana* aerial parts (CSH) obtained using hexane solvent reduced 65.5% of Tulahuen infection in L929 host cells whereas the other purified extracts and fractions from this specie reduced ≤10%. Natural products from fruit pulp of *C. cujete* have no trypanocidal effect at a concentration of 10 µg/mL. Likewise, crude extracts and studied fractions from leaves of *M. glabra* and from *R. longifolia* were inactive against this protozoan. On the other hand, crude extract of *P. tectoniifolium* obtained using ethanol as solvent (PTFrE) largely reduced (83.7%) *T. cruzi* infection in vitro. Taken together, our findings demonstrated that 3 out of the 36 botanic natural products presently studied (including 7 crude extracts and 29 fractions, Table 1), namely AFfPD, CSH, and PTFrE were able to reduce (>65%) the infection of *T. cruzi*-parasitized cell cultures while the others were found inactive or slightly active at a fixed concentration of 10 µg/mL, following a standardized protocol as previously recommend to identify a promising hit drug candidate for Chagas disease [26].

The next step was the determination of the potency of the two best natural products (AFfPD and PTFrE) upon the intracellular forms of *T. cruzi*, using dose-response assays. Our data demonstrated that AFfPD and PTFrE displayed a considerable effect against the proliferative intracellular forms of *T. cruzi*, reaching EC_50_ values of 9.3 ± 1.9 and 12.6 ± 1.9 μg/mL, respectively (Table 2). The cytotoxicity of these biomolecules on mammalian cells was also investigated. In these studies, the host cells (L929 cell lines) were incubated with increasing concentrations (up to 200 μg/mL) of both natural products as well as Bz. The results shown in Table 2 demonstrate that although the reference drug was not toxic up to the higher tested concentration, AFfPD and PTFrE displayed a toxic profile presenting LC_50_ values of 12.5 ± 1.0 and 15 ± 3.0 μg/mL, respectively, leading to lower selectivity indexes (SI ≤ 1.3) as compared to Bz (Table 2).

In addition, the activity of both natural products was also evaluated against bloodstream trypomastigotes (BT), another relevant form of *T. cruzi* in mammalian hosts (Table 2). BT of Y strain (DTU II, partially resistant to Bz [27] were treated for 2 and 24h using increasing compound concentrations, up 50 µg/mL. While Bz was inactive at a short time-period of incubation (2 h), AFfPD presented a fast killer profile, displaying EC_50_ = 38.7 µg/mL. After 24 h of treatment, AFfPD sustained a higher trypanosomicidal effect as compared to Bz, given by its lower EC_50_ and EC_90_ values (2.2 ± 1.0 µg/mL and 7.9 ± 2.2. µg/mL, respectively), being about five- and three-fold more active against BT than Bz. PTFrE displayed a moderate trypanocidal activity (EC_50_ = 38.8 ± 2.1 µg/mL). As cardiomyopathy is one of the main chronic symptoms of CD, next, the cardiotoxicity profile was assessed using primary cultures of mouse cardiac cells (CC) incubated with increasing concentration of both biomolecules and Bz. After 2h of incubation with both extracts, no evident cardiac alterations related to cellular density and morphology was observed by light microscopy. However, after 24 h of incubation, AFfPD showed cardiotoxicity while PTFrE was not toxic, displaying LC_50_ values of 15.2 ± 2.7 µg/mL and 124.2 ± 6.8 µg/mL, respectively (Table 2), also corroborating previous findings using macrophages (11)

The present trypanocidal results validated previous studies using the dichloromethane fraction of *A. fasciculata* var. *fasciculata* upon promastigotes of *Leishmania amazonensis* [11]. In fact, previous report demonstrated that other withanolides also display trypanocidal activity against epimastigote and trypomastigote forms of *T. cruzi* in vitro [28], justifying further studies using this class of botanic source as novel anti-parasitic agents.

### 2.2. Purified Substances

**Figure 2:**
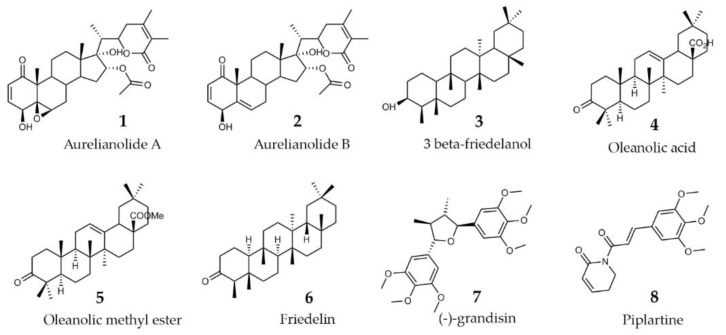
Chemical structures of the studies purified substances.

As literature reported the anti-trypanosomatid activity of withanolides isolated from *A. fasciculata* var. *fasciculata* (Aurelianolides A and B [11]) and of plant extracts purified from *Clusia* sp. [29] and *Piper* sp. [24], the trypanocidal effect of eight purified substances from the dichloromethane fraction of *A. fasciculata* var. *fasciculata*, and from crude extracts of *C. studartiana* and *Piper* sp. was further investigated (Figure 2). 

To ascertain the antiparasitic action of these biomolecules, phenotypic studies were carried out against intracellular forms using a concentration of 10 μM, which is the threshold considered for anti-*T. cruzi* hit [26 Aurelianolide A and B (compound **1** and **2**) from *A. fasciculata* var. *fasciculata* greatly reduced (>90%) the infection of L929 cell lines (Tulahuen-B gal strain), exhibiting similar activity as Bz (Table 3). Additionally, compound **8** from *P. tuberculatum* displayed moderate activity, decreasing the culture parasitization approximately 50% (Table 3).

**Figure 3:**
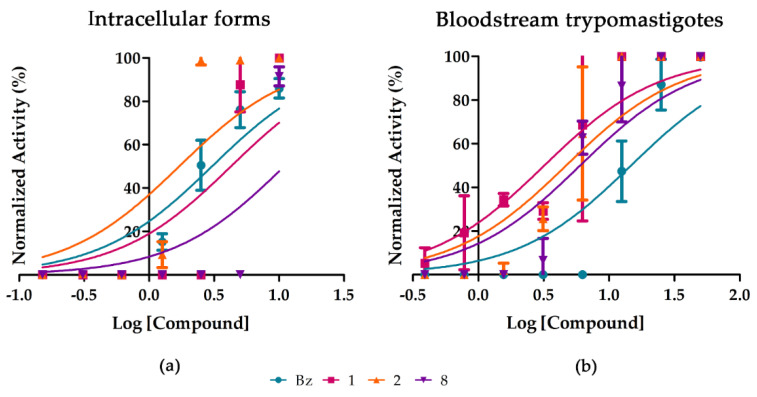
Activity of compounds **1**, **2** and **8** against intracellular forms of Tulahuen strain (**a**) and on bloodstream trypomastigotes of Y strain (**b**) after 24 and 96 h of incubation, respectively. The X-axis shows log of compound molar concentrations (M), and Y-axis shows the normalized activity.

Next, dose-response studies were conducted with the three more active purified substances. Our findings confirmed their antiparasitic activity upon the different parasite forms (intracellular amastigotes and bloodstream trypomastigotes) and DTUs relevant for human infection (DTUs VI and II, Tulahuen and Y strains, respectively). Compound **2** (Aurelianolide B) was the most potent and selective on intracellular forms of Tulahuen strain (Figure 3a), being twice as active as the reference drug, reaching EC_50_ value of 1.6 ± 0.4 μM and selectivity index of 17.1. Compound **1** (aurelianolide A) gave an antiparasitic activity (EC_50_ of 4.6 ± 1.3 μM) in the same range as Bz (EC_50_ of 3.0 ± 0.6 μM) while compound **8**, from *P. tuberculatum* reached value of 8.1 ± 0.9 μM. Additionally, compounds **1**, **2** and **8** were highly effective upon bloodstream trypomastigotes of *T. cruzi* (Y strain), with EC_50_ and EC_90_ ranging from 5.38 ± 0.3 to 5.72 ± 1.3 μM and 8.33 ± 3.9 to 14.44 ± 5.8 μM, respectively, being at least two-fold more active than Bz (Figure 3b and Table 4). The compounds toxicity was evaluated towards two different mammalian cell types: L929 cell lines and primary cultures of heart cells (Table 4). The results demonstrated a mild toxicity towards mammalian cells, leading to SIs ranging from 17.1 to 2.9. Compound **1** exerted the higher cardiotoxicity, exhibiting LC_50_ = 16.36 ± 1.3 µM (Table 4). 

Considering the anti-*T. cruzi* activity of these three purified compounds, physicochemical parameters, Lipinski’s rule of five (Table 5), and the absorption, distribution, metabolism, excretion, and toxicity (ADMET) properties were predicted (Table 6) using pKCSM tool. Bz and compound **8** did not infringe Lipinski’s rule of five, predicting a good oral absorption. As reported by Lima et al. (2018) [11], both withanolides presented molecular weight just over 500 and had acceptors above 5 (Table 5). 

As also reported by Lima et al. (2018) [11], in silico ADMET analysis (Table 6) predicted a good permeability on intestinal Caco-2 cells, with values close or above 9, and high intestinal human absorption (above 87%) for compounds **1**, **2** as we also presently found for compound **8,** which is not expected to act on P-glycoprotein. As previously depicted for both withanolides [11], compound **8** is not likely to cross the blood-brain barrier (BBB), has limited access to the central nervous system (CNS) and is likely to be metabolized by CYP1A2. Regarding toxicity parameters, as previously predicted for both compounds **1** and **2** [11] and unlike Bz, compound **8** has no mutagenic nor hepatotoxicity profile. 

Presently, we investigated the cytotoxicity and anti-*T. cruzi* activity of 36 natural products such as extracts, fractions and 8 purified compounds obtained from the Brazilian flora. The bulk of our results brings important contributions to the search of novel drug candidates for the treatment of Chagas disease, a neglected illness demanding new therapeutic options [30]. Although active against intracellular and bloodstream trypomastigote forms, the toxicity toward mammalian host cells of AFfPD (*A. fasciculata* var. *fasciculata*), CSH (*C. studartiana*) and PTFrE (*P. tectoniifolium*) resulted in quite low selective indexes. As the toxic profile could be derived from the highly heterogeneous composition of the extracts and fractions, we further investigated the activity of eight purified substances isolated from *A. fasciculata* var. *fasciculata*, *C. studartiana*, *P. tectoniifolium* and *P. tuberculatum.* In fact, previous literature data also reported promising anti trypanosomatid effect, of some of these biomolecules, specially towards *Leishmania* sp. [11,24,29]. Three of these compounds showed encouraging results. The piplartine isolated from *P. tuberculatum* displayed high activity against both intracellular and bloodstream forms of *T. cruzi* in vitro (EC_50_ = 8.1 and 5.4 μM, respectively) corroborating previous findings reporting anti-*T. cruzi* activity of other biomolecules isolated from different *Piper* species. According to Vieira et al. (2018) [31], piplartine may induce an imbalance of enzymes related to redox metabolism in some trypanosomes, due to the oxidation process of several amino acid residues. Likewise, other natural metabolites found in *Piper* species, such as the volatile monoterpene [32] linalool (one of the main constituents of an essential oil of *P. aduncum)*, showed an outstanding effect against cell-derived trypomastigotes (EC_50_ = 0.31 μg/mL), although still with a limited selectivity index of 2.9 [33]. Similarly, the furofuran metabolite from *P. jericoense* was active in vitro, with selective index of 4.7; besides being able to reduce the parasitemia levels in *T. cruzi*-infected mice [34]. The authors describe that this lignan induced alterations on the parasite structure, but did not alter the energetic metabolism [34]. 

The withanolides, aurelianolide A and aurelianolide B, were able to decrease the parasite load of L929 cell lines infected by Tulahuen strain, revealing a high potency not only intracellular forms (EC_50_ of 4.6 and 1.6 μM, respectively) but also upon bloodstream trypomastigotes (Y strain) with quite similar EC_50_ values (5.68 and 5.72 μM, respectively), being twice more potent than the reference drug. Our findings are in accordance to those reported by Lima et al. (2018) [11], which evaluated the antileishmanial effect of fractions and purified withanolides from *A. fasciculata* leaves [11], isolated as described by Almeida-Lafetá (2010) [35]. The dichloromethane fraction and both aurelianolide A and B were active against promastigotes of *L. amazonensis* and both withanolides also presented high potency upon infected macrophages (EC_50_ = 2.3 μM and 6.43 μM, selectivity indexes = 5.6 and 2.0, respectively). Our present findings related to the selectivity indices of the studied extracts and purified substances are far from ideal to consider them antitrypanosomal agents, and additional analysis are desirable for molecular optimization aiming improve the therapeutic window.

In silico ADMET analysis of aurelianolide A and aurelianolide B were previously evaluated by Lima and colleagues (2018) [11] and presently we compared the withanolides prediction with the data of compound **8** (piplartine), using the pkCSM tool. Lipinski’s rule of five can predict drug absorption and permeability, from parameters which values are 5 or multiples of 5 [36]. Aurelianolide A and aurelianolide B violated the parameters of molecular weight and acceptors, as reported by Lima et al. [11]. In addition, ADMET parameters revealed that compounds **1**, **2** and **8** are water-soluble and likely to be permeable to intestinal cells and the human gut. These compounds are also expected to have low toxicity to the CNS due to their low permeability and are poorly metabolized by liver enzymes. The toxic prediction profile suggests that the compounds **1**, **2** and **8** are safe for oral administration (at low doses), but these results must be confirmed by in vivo studies. In fact, although computational analysis allows a fast and low cost of ADMET properties, additional experimental tests are needed to confirm these predictions. The toxic effect of aurelianolide A and aurelianolide B metabolites can be correlated to presence of α, β-unsaturated ketone in ring A, as well as the conjugated ketone in the lactone ring. Both electrophilic moieties display an important role for oxidative damage process and reacting with proteins by thiol alkylation and oxidation [37].

Altogether, our results showed that natural products, especially isolated and purified compounds as we presently evaluated, merits to be widely explored, as they provide important insights related to novel drug candidates hits for therapy of tropical neglected diseases.

## 3. Materials and Methods

### 3.1. Botanical Material and Extracts and Purified Fractions

All plants species obtained from the Brazilian flora were provided by Instituto de Tecnologia em Fármacos da Fundação Oswaldo Cruz (Farmanguinhos/Fiocruz, Rio de Janeiro, Rio de Janeiro, Brazil. Preparation of the extracts and fractions: Leaves, branches, fruit pulp or inflorescence of plant species were dried in an oven with circulating air at 40 °C. The dried material was reduced to small fragments and submitted to extraction by dynamic maceration, at room temperature with hexane, ethanol or methanol. The solvent was removed under low pressure to obtain the dry extracts. The samples were kept in vacuum desiccator to remove small amounts of remaining solvents. Part of these extracts were solubilized in methanol:water 1:9 (*v*/*v*) and fractionated by liquid-liquid partition, using solvents of increasing polarities sequentially: hexane, dichloromethane, chloroform, ethyl acetate and butanol. The fractions resulting from the partition and the aqueous residue were concentrated under reduced pressure or lyophilized. The chemical profile of the extracts and fractions were obtained by HPLC. For the isolation of compounds different chromatographic techniques of phytochemistry were used. Structure elucidation of pure compounds were determined by ^1^H and ^13^C NMR and by comparison with data from the literature. All the studied species are registered in the Brazilian Genetic Patrimony (SISGEN) under the number AB5D582. The samples were prepared as stock solutions in dimethyl sulfoxide (DMSO; Sigma-Aldrich, St. Louis, MO, United States) and used as final working concentrations, never exceeding 0.6%, which means no toxic effect for the mammalian cells and parasites [38].

### 3.2. Mammalian Cell Cultures

Primary cardiac cell cultures (CC) were obtained from Swiss Webster mice embryo hearts (18 gestation days) as described by Meirelles et al. [39] and were used for toxicity assays. After purification, the cardiac cells were seeded (0.5 × 10^5^ cells/well) in 96 wells microplates previously coated with 0.01% gelatin. The cardiac cultures were maintained in Dulbecco’s Modified Eagle’s Medium supplemented with 6% of FBS, 2.5 mM CaCl_2_, 1 mM L-glutamine, 1mM penicillin and 2% chicken embryo extract. Mice fibroblast of the line NTCL clone 929 (L929) were used for toxicity and anti-*T. cruzi* activity assays [40]. L929 cells were seeded (4 × 10^3^ cells/well) in 96 well microplates and kept in RPMI-1640 medium (Sigma-Aldrich, Missouri, EUA) supplemented with 10% FBS, 1% L-glutamine and 1% penicillin. The cell cultures were maintained at 37 °C at 5% CO_2_.

### 3.3. Mammalian Cell Toxicity

CC and L929 cells were incubated for 24 and 96 h with increasing concentrations of the compounds (serially diluted (7-fold), 1:2 up to the solubility limit: 0–400 µg/mL for extracts/fractions and 400 µM for purified substances, while for Bz was 0–750 µM) diluted in Eagle’s medium or RPMI-1640 medium supplemented with 1% L-glutamine. Cell viability was assessed using PrestoBlue (for assay with CC) and AlamarBlue (for assays with L929) with fluorescence and absorbance readouts (560–590 and 570–600 nm, respectively) in a spectrophotometer (Tunable microplates reader VersaMax; Molecular Devices). Additionally, cell morphology aspects were evaluated in a light microscope, as well as the contractability, density and cytoplasmic vacuolization. Cell viability rates were then determined by the LC_50_ values that correspond to the compound concentration able to reduce in 50% the viability of the cell population [41]. 

### 3.4. Trypanocidal Activity

Bloodstream trypomastigotes (BT) of the *T. cruzi* Y strain (DTU II) were obtained by cardiac punction from Swiss Webster mice during the parasitemia peak and the purified parasites were resuspended in Dulbecco’s modified Eagle medium (DMEM) (Cultilab) supplemented with 10% fetal bovine serum (FBS) [39]. BT of Y strain were incubated for 24 h at 37 °C in RPMI-1640 medium supplemented with 5% FBS and increasing concentrations (serially diluted (7-fold) of the compounds and Bz (0–50 µg/mL for extracts and fractions and 0–50 µM for purified compounds and Bz). After the incubation, the indexes of parasite lyses were determined by the quantification of the motile and live parasites by light microscope quantification using Neubauer chamber to determine the EC_50_ and EC_90_ values [42,43]. 

Culture derived trypomastigotes of Tulahuen strain (DTU VI), transfected with the β-galactosidase gene from *Escherichia coli*, were collected from the supernatant of previously infected L929 fibroblasts [40]. Trypomastigotes were used to infect L929 cultures at a ratio of 10:1 (parasites/host cell) to determine activity against intracellular forms. After 2 h of interaction, the cultures were washed for the removal of remaining parasites and incubated for 48 h to ensure the establishment of the infection. On the first set of assays, the compounds were added in a fixed concentration (10 µg/mL or 10 µM for the extracts and purified fractions, respectively) that corresponds to EC_90_ of Bz. The non-infected cells (controls) were treated with DMSO and Bz and were also evaluated. The compounds that reached the same or higher activity than Bz were later evaluated in assays with increasing concentrations (serially diluted (7-fold): 0–50 µg/mL for extracts and fractions and 0–50 µM for purified compounds and Bz) for the determination of the EC_50_ and EC_90_ values (minimal concentration able to reduce to 50% and 90% of the infection index in cultures). In both assays the cultures were maintained at 37 °C for 96 h to enable the calculation of the inhibition percentage on the infection [40]. Thereafter, 500 μM of substrate CPRG (chlorophenol red-β-D-galactopiranosideo) in 0.5% Nonidet P40 (Sigma-Aldrich, St. Louis, MO, United States) were added to the in vitro system. The plates were incubated for 18 h at 37 °C and, following, the absorbance was measured at 570 nm. The results were expressed as the percentage inhibition of the infection by *T. cruzi*, hence, comparing the data of optical density of the tested compounds obtained from the non-infected cell cultures, the infected ones and those treated only with DMSO or Bz [42,43]. All the assays were conducted in duplicate at least three times.

### 3.5. In Silico ADMET Properties

Absorption, distribution, metabolism, excretion, and toxicity (ADMET) and Lipinski’s rule of five properties of compounds **1**, **2** and **8** and BZ were accessed using the Predicting Small-Molecule Pharmacokinetic and Toxicity Properties (pKCSM) approach, which uses graph-based signatures to develop predictive ADMET [44,45].

### 3.6. Statistical Analyses

The statistical analysis was performed individually for each assay using Student’s *t*-test and ANOVA with the level of significance set at *p* ≤ 0.05.

### 3.7. Ethics

All animal studies were carried out in strict accordance with the guidelines established by the FIOCRUZ Committee of Ethics for the Use of Animals (CEUA L038-2017).

## Figures and Tables

**Table 1:** Botanic species, corresponding part of the plant, applied solvent and anti-*T. cruzi* activity profile (% of infection reduction of L929 cell lines) of the natural products and of the reference drug (benznidazole) under a fixed concentration of 10 µg/mL upon intracellular forms of *T. cruzi* (Tulahuen strain).

Family/Species	Part of the Plant	Compound/Extract	Solvent	% of Infection Reduction after 96 h of Incubation
-	-	Benznidazole	-	90.5 ± 5.7
Solanaceae *Aureliana fasciculata* var. *fasciculata*	Leaves	AFFMeOH ^1^	Methanol	6.5 ± 9.0
		AFfPD	Dichloromethane	100.0
		AFFHex	Hexane	12.3 ± 5.0
		AFFAcoEt	Ethyl acetate	11.9 ± 6.1
		AFFBuOH	Butanol	11.1 ± 6.3
		AFFAquo	Aqueous partition	10.3 ± 5.6
Clusiaceae *Clusia studartiana*	Leaves	CSH-F ^1^	Hexane	6.8 ± 6.3
	Aerial parts	CSH ^1^	Hexane	65.5 ± 23.3
		CSE ^1^	Ethanol	0.0
		CSTE-C	Chloroform	5.7 ± 8
		CSTE-Ac	Ethyl acetate	3.9 ± 5.8
		CETE-Bu	Butanol	2.8 ± 5.8
		CSTE-Aq	Aqueous partition	10.0 ± 6.0
Bignoniaceae *Crescentia cujete*	Fruit pulp	CCPEH	Hexane	2.9 ± 0.8
		CCPEC	Chloroform	10.9 ± 3.5
		CCPEAC	Ethyl acetate	0.0
		CCPEBU	Butanol	0.0
		CCPEA	Aqueous partition	1.3 ± 1.8
		CCPEACN + MEOH	Acetonitrile/methanol	0.2 ± 0.5
Malpighiaceae *Malpighia glabra*	Leaves	MGE ^1^	Ethanol	9.4 ± 3.5
		MGEH	Hexane	8.8 ± 5.3
		MGED	Dichloromethane	2.5 ± 3.6
		MGEAc	Ethyl acetate	4.6 ± 7.3
		MGEB	Butanol	10.4 ± 4.3
		MGEAq	Aqueous partition	1.0 ± 1.0
Piperaceae *Piper tectoniifolium*	Inflorescence	PTFrE ^1^	Ethanol	83.7 ± 15.0
		PTFrEPH	Hexane	18.9 ± 5
		PTFrEPD	Dichloromethane	19.4 ± 7.2
		PTFrEPAc	Ethyl acetate	22.1 ± 10.3
		PTFrEPB	Butanol	21.3 ± 6.0
Clusiaceae *Rheedia longifolia*	Leaves	RLFM ^1^	Methanol	16.0 ± 2.8
		RLFMH	Hexane	12.5 ± 10.2
		RLFMD	Dichloromethane	14.2 ± 7.0
		RLFMAc	Ethyl acetate	8.5 ± 9.7
		RLFMBu	Butanol	12.5 ± 15.8
		RLFMAq	Aqueous partition	12.0 ± 15.8

^1^ Crude extracts.

**Table 2:** Activity (50% effective concentration, μg/mL) of the natural products against intracellular and bloodstream forms of *T. cruzi* (EC_50_), mammalian cell’s toxicity (L929 cell lines and cardiac cell cultures-LC_50_) and respective Selectivity Indexes (SI).

Cp	Intracellular Forms (Tulahuen Strain)			Bloodstream Trypomastigotes (Y Strain)					
	96 h			2h		24 h			
	EC_50_	LC_50_^a^	SI	EC_50_	EC_90_	EC_50_	EC_90_	LC_50_^b^	SI
Bz	3.8 ± 1.8	> 200	> 52	>50	>50	10.2 ± 0.3	23.3 ± 0.6	>750	>83
AFfPD	9.3 ± 1.9	12.5 ± 1.0	1.3	38.7 ± 1.2 *	>50	2.2 ± 1.0 *	7.9 ± 2.2 *	15.2 ± 2.7	7.1
PTFrE	12.6 ± 1.7	15 ± 3.0	1.2	>50	>50	38.8 ± 2.1	47.4 ± 1.2	124.2 ± 6.8	3.2

^a^ L929 cell lines; ^b^ cardiac cell cultures; * *p* < 0.05 determined by ANOVA. EC_50_ and LC_50_ value: 50% effective concentration for parasites and host cells, respectively.

**Table 3:** Data (species and metabolites) and anti-*T. cruzi* (Tulahuen strain) activity at 10 μM after 96 h of incubation (% reduction of L929 cell line infection) with the eight tested plant purified substances.

Cp	Botanical Species	Chemical Compounds	% of Infection Reduction
Bz	-	Nitro-heterocyclic	92 ± 7
1	*Aureliana fasciculata*	Aurelianolide A	95.2 ± 0
2		Aurelianolide B	91 ± 16
3	*Clusia studartiana* var. *fasciculata*	3 beta-friedelanol	21 ± 10
4		Oleanolic acid	20 ± 18
5		Oleanolic methyl ester	36 ± 24
6		Friedelin	0
7	*Piper tectoniifolium*	(-)-grandisin	25 ± 36
8	*Piper tuberculatum*	Piplartine	54.1 ± 29

**Table 4:** Activity of the purified substances against intracellular and bloodstream forms of *T. cruzi* (EC_50_ µM) mammalian cell’s toxicity (L929 cell lines ^1^ and cardiac cell cultures ^2^-LC_50_, μM) and corresponding Selectivity Indexes (SI).

Cp	Intracellular Forms			Bloodstream Trypomastigotes				
	96 h			2 h	24 h			
	EC_50_	LC_50_ ^1^	SI	EC_50_	EC_50_	EC_90_	LC_50_ ^2^	SI
Bz	3 ± 0.6	>200	>66.6	>50	13.13 ± 3.9	31.29 ± 8.8	>750	>57.1
1	4.6 ± 1.3	42.1 ± 28.3	9.15	>50	5.68 ± 2.3	8.33 ± 3.9	16.36 ± 1.3	2.9
2	1.6 ± 0.4	27.36 ± 1	17.1	>50	5.72 ± 1.3	9.25 ± 3.2	30.89 ± 3.8	5.4
8	8.1 ± 0.9	73.1 ± 75.5	9	>50	5.38 ± 0.3	14.44 ± 5.8	33.15 ± 5.9	6.2

^1^ L929 cell lines, ^2^ cardiac cell cultures.

**Table 5:** Physicochemical parameters and Lipinski’s rule of five using pkCSM tool.

Property	Reference	Bz	1	2	8
Molecular Weight	≤500	260.253	528.642	512.643	317.341
LogP	≤5	1.1077	3.037	3.8258	2.0407
Acceptors	≤5	5	8	7	5
Donors	≤10	1	2	2	0

**Table 6:** In silico ADMET using pkCSM tool.

Property	Reference	Bz	1	2	8
ABSORPTION					
Water solubility (log mol/L)	-	−2.782	−4.994	−5.155	−3.7
Caco2 permeability (log cm/s)	>0.9	0.542	0.85	0.894	1.419
Intestinal absorption (human, %)	<30% is poorly	75.834	92.85	87.412	97.785
Skin Permeability (log Kp)	>−2.5 is low	−2.768	−3.143	−3.564	−3.229
DISTRIBUTION					
P-glycoprotein substrate	No	Yes	Yes	Yes	No
P-glycoprotein I inhibitor	No	No	Yes	Yes	No
P-glycoprotein II inhibitor	No	No	Yes	Yes	No
VDss (human) (log L/kg)	Low is <−0.15, High is >0.45	−0.364	0.174	−0.011	−0.067
Fraction unbound (human)	-	0.299	0.061	0	0.255
BBB permeability (log BB)	Poorly is <−1, High is >0.3	−0.49	−0.406	−0.214	−0.47
CNS permeability(log PS)	Penetrate is >−2, Unable is <−3	−2.731	−2.757	−2.501	−2.947
METABOLISM					
CYP2D6 substrate	No	No	No	No	No
CYP3A4 substrate	-	No	Yes	Yes	No
CYP1A2 inhibitior	No	No	No	No	Yes
CYP2C19 inhibitior	No	No	No	No	No
CYP2C9 inhibitior	No	No	No	No	No
CYP2D6 inhibitior	No	No	No	No	No
CYP3A4 inhibitior	No	No	No	No	No
EXCRETION					
Total Clearance (log ml/min/kg)	-	0.539	0.335	0.416	0.266
TOXICITY					
AMES toxicity	No	Yes	No	No	No
Max. tolerated dose (human-log)	Low is ≤0.477, High is >0.477	0.733	−0.878	−0.875	0.573
hERG I inhibitor	No	No	No	No	No
hERG II inhibitor	No	No	No	No	No
Oral Rat Acute Toxicity (LD_50_)	-	2.251	3.672	3.066	2.528
Oral Rat Chronic Toxicity	-	1.594	2.12	1.957	2.657
Hepatotoxicity	No	Yes	No	No	No
Skin Sensitisation	No	No	No	No	No
*T. pyriformis* toxicity (log ug/L)	>−0.5 is toxic	0.285	0.286	0.294	0.597
Minnow toxicity (log mM)	<−0.3 is toxic	0.803	2.529	1.526	1.433

## Data Availability

Data supporting reported results can be found at the Laboratorio de Biologia Celular do Insittuto Oswaldo Cruz and at the Laboratório de Tecnologia para Biodiversidade em Saúde/LDFito, Instituto de Tecnologia em Fármacos (Farmanguinhos), Fundação Oswaldo Cruz (FIOCRUZ).
